# Genotype-by-Environment Interaction Analysis for Quantity and Quality Traits in Faba Beans Using AMMI, GGE Models, and Stability Indices

**DOI:** 10.3390/plants12213769

**Published:** 2023-11-04

**Authors:** Vasileios Greveniotis, Elisavet Bouloumpasi, Stylianos Zotis, Athanasios Korkovelos, Dimitrios Kantas, Constantinos G. Ipsilandis

**Affiliations:** 1 Hellenic Agricultural Organization Demeter, Institute of Industrial and Forage Crops, GR-41335 Larissa, Greece; 2 Department of Agricultural Technology, Technological Educational Institute of Western Macedonia, GR-53100 Florina, Greece; 3 Department of Agricultural Biotechnology and Oenology, International Hellenic University, GR-66100 Drama, Greece; elisboul@abo.ihu.gr; 4 Directorate of Water Management of Thessaly, Decentralized Administration of Thessaly—Central Greece, GR-41335 Larissa, Greece; athanasios.korkovelos@apdthest.gov.gr; 5 Department of Animal Science, University of Thessaly, Campus Gaiopolis, GR-41500 Larissa, Greece; dkantas@uth.gr; 6 Regional Administration of Ionian Islands, GR-49100 Corfu, Greece; ipsigene@gmail.com

**Keywords:** yield, stability, stability index, selection, adaptability, faba beans, genotype, environment interaction, additive main effects and multiplicative interaction, genotype plus genotype-by-environment

## Abstract

Faba beans are considered one of the most important crops for animal feed. The genotype × environment interaction (GEI) has a considerable effect on faba bean seed production. The objectives of this study included assessing multiple locations and genotypes to understand how various ecosystems and faba bean genotypes relate to one another, and suggesting the ideal climatic conditions, crop management system, and genotypes so that they are carefully chosen for their stability. A 2-year experiment was conducted in order to define the stability across four environments based on stability indices for certain characteristics: moisture (%), ash content (%), crude protein content (%), crude fat (%), total starch (%), and crude fiber content (%). Statistically significant differences indicated that GEIs were present. The heritability was generally high for qualitative traits in comparison with quantitative traits. The crude protein content, plant height, and thousand-seed weight were all positively correlated with the seed yield; however, the other qualitative variables were adversely correlated. The crude protein content of the cultivar Tanagra displayed a high stability index, followed by Ste1. Under conventional management, Tanagra demonstrated high values for the seed yield in Giannitsa and Florina. Ste1 and Ste2 are particularly promising genetic materials that showed high values under low-input conditions. The best genotypes to use and the most favorable environments/types of cultivation were the Tanagra cultivar, followed by the Ste2 genotype, according to the additive main effects and multiplicative interaction (AMMI) and genotype plus genotype-by-environment (GGE) biplot models. Earliness showed significant heritability values and very high stability indices, again indicating qualitative behavior according to genetic parameters. With the exception of the number of pods per plant, which demonstrated low heritability while having excellent index values, traits like seed yield showed relatively low-stability-based heritability values. Global efforts aimed at improving the genetics of faba beans might benefit from genotypes that exhibit consistent yields in various conditions.

## 1. Introduction

Broad beans, or faba beans (*Vicia faba* L.), are known as a grain legume crop for both human consumption and animal feed [1]. Faba bean is one of the earliest domesticated food legumes, which was found as a cultivated plant in ancient times [2], especially in the Mediterranean basin [3]. It is reported to have 3% saturated sugars, 6.5% oligosaccharides, and about 27–34% proteins and starch at about 45% of the total dry mass [4]. As far as animal nutrition is concerned, tannins, vicine, and convicine are found in faba beans and are considered to have antinutritional effects in the diet of various monogastric animals [5,6,7]. Divicine and isouramil are considered toxic to humans with a widespread genetic mutation (G6PD) [8]. The same authors also described the seed composition in detail. The main traits of faba beans were described by Loss and Siddique [9], while strong genotype × environment (GxE) interactions concerning yield were reported by Fox et al. [10] and Toker [11].

In a continuously changing environment, the stability of the performance of many traits is considered very important in order to enhance food availability and security [12,13]. Regarding stability, many analyses have been conducted, including isozymes or PCR-based data [14,15,16,17], and the sequence-specific amplification polymorphisms (SSAPs); target region amplification polymorphisms (TRAPs); random amplification of polymorphic DNAs (RAPDs); restriction fragment length polymorphisms (RFLPs); amplified fragment length polymorphisms (AFLPs); and the newer markers, such as the start-codon-targeted (SCoT) polymorphism, are used in order to analyze the genetic variability of *Vicia* species and *V. faba* L. populations [7,18]. Stability indicates that the genotype positively responds to any improvement in environmental conditions and can perform above the mean in different locations [19]. This behavior is of great importance for both plant breeders and farmers. In parallel, multi-location field experiments were extensively used in order to improve the adaptability and reduce the environmental effects on genotype behavior [20], especially for yield, which is significantly affected by ecological conditions in terms of stability and adaptation [21].

The genotype (G), environment (E), and genotype × environment interaction (GEI) all have a significant impact on the seed yield, which is an extremely complex trait [11]. Under various environmental situations, the GEIs lead genotypes to respond differently [22]. Given that GE interaction decreases the correlation between phenotypic and genotypic values across locations, it is crucial for breeders [23]. It also has an impact on selecting appropriate test conditions, allocating resources within breeding programs, and selecting breeding germplasm and tactics [24,25]. In the case of breeding legumes, GE interaction presents a difficulty because prior research has indicated that a significant amount of the variation in seed production in pulse crops, including faba bean, is influenced by both the environment (E) and GE interaction [24,25]. To comprehend and explain the GEIs, a variety of statistical techniques were devised [26,27]. Among the different currently available methods, the additive main effect and the multiplicative interaction (AMMI) analysis [28] and the genotype plus genotype-by-environment (GGE) biplot [29] are two of the methods that are most frequently used in multi-environmental trial analysis because they offer more precise estimates and simple interpretations of the GEIs using understandable graphical tools. There are various benefits to using the AMMI or GGE biplot to explain GEIs. However, there are also drawbacks [30] and their combinations with stability indices allow for the selection of genotypes for multiple traits [31,32,33,34,35,36,37]. The genotype main effect plus genotype per se × environment (GGE) biplot enables simultaneous analysis of genotypes by considering high-yielding ability and stability. The combination of these two concepts is known as the “ideal genotype” [38,39]. GGE biplots were extensively used in other species in order to define stable cultivars across environments, revealing the best-adapted genotype [31,32,33,34,35,36,37]. They were also used in a few studies regarding the selection of stable faba bean genotypes across environments in Greece [40], Ethiopia [20,41,42], and Lebanon [43]. Thus, the GGE biplot model is considered a strong tool for the effective analysis of the multi-environment data structure in breeding [41]. The purpose of this study was (a) to understand the relationship between faba bean (*Vicia faba* L.) genotypes, various ecosystems, and GEI in order to (b) identify stable and high-yielding genotypes to specific environments or across environments for future breeding in various environments using the stability index tool [44] based on seed quality and chemical composition parameters. Another objective of this study was to examine how the GEI influenced the seed yield, seed quality parameters, and seed composition.

## 2. Results

### 2.1. Stability Index Measurements and ANOVA

Table 1 presents the ANOVA results for the quantitative and qualitative traits of faba beans. In all cases, there were statistically significant differences, but of great importance were the GxE interactions, which were very significant at *p* < 0.01 level, except for earliness and crude protein (significant at *p* < 0.05 significance).

In Table 2, earliness, starch content, and plant height showed indices over 1000, especially earliness, which reached 4000 in the Trikala area and conventional treatment (almost 3500 with the low-input treatment), indicating high stability capacity for these traits. For seed yield, greater indices were found in the Kalambaka area (79 and 302 in conventional and low-input conditions, respectively). Trikala and Kalambaka also showed high indices for plant height (over 1000).

Table 3 presents the stability indices concerning genotypes. Ste1 and Tanagra showed some extreme index values for earliness that were even close to or above 5000, followed by Ste2, while Polycarpe showed much lower indices. For seed yield, the improved cultivar Tanagra showed an unstable behavior and low indices in low-input conditions, but for protein content, Tanagra showed the best results.

More detailed results are presented in Table 4. For earliness, extreme values were found in many areas for the genotypes tested. In Trikala and Kalambaka, especially Ste1, followed by the cultivar Tanagra, showed some index values that ranged from 12,000 to almost 23,000 under conventional treatment. For the seed yield, Tanagra showed high values in Giannitsa (over 700) and Florina under conventional treatment. Ste1 and Ste2 are very promising genetic materials that showed high values in Trikala and Kalambaka under low-input conditions. Tanagra and Ste1 demonstrated high crude protein content levels for both treatments (low input and conventional), especially for Giannitsa and Florina.

### 2.2. Heritability Estimations

Table 5 presents genetic parameters for all traits. All qualitative traits showed high heritability values based on the stability index, with over 90 and, in some cases, near 100, except for water content. Earliness, although considered a quantitative trait, showed high heritability and, in combination with Table 2, Table 3 and Table 4, very high indices. Quantitative traits like seed yield showed relatively low heritability values but at a satisfactory level (over 87), except for the number of pods per plant, where heritability was very low (close to 30), while from Table 2, Table 3 and Table 4, this trait showed satisfactory index values.

### 2.3. Correlations between Traits

In Table 6, the correlations between all traits are presented. The seed yield was positively correlated with the thousand-seed weight (0.938), plant height (0.359), and crude protein content (0.291), but negatively correlated with the rest of the qualitative traits, like starch content (−0.319).

The number of pods per plant seemed not to affect the rest of the qualitative or quantitative traits (usually non-significant correlations, but positively correlated with starch content).

### 2.4. AMMI and GGE Biplots

The AMMI and GGE biplot data are presented in Figure 1 and Figure 2 and Supplementary Figures S1–S9.

**Figure 1:**
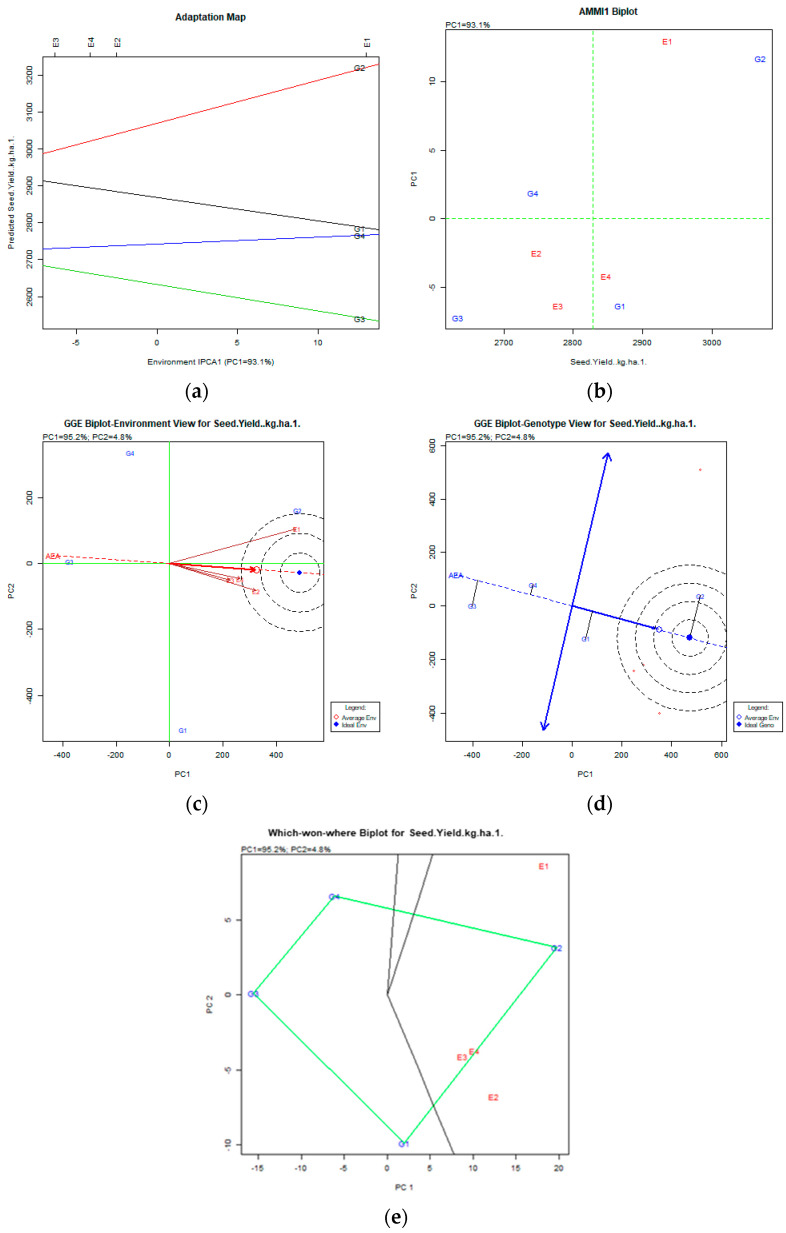
Seed yield (kg ha^−1^) stability analysis based on (**a**) AMMI adaptation map, (**b**) AMMI1 biplot, (**c**) environmental stability GGE biplot, and (**d**) genotypic stability GGE biplot. The genotypes closer to the ideal genotype are more desirable. (**e**) Which-won-where GGE biplot for specific adaptability of genotypes over environments.

After the stability analysis using AMMI and GGE biplot software (PB tools v1.4 free version), for the seed yield trait, the variability explained by the AMMI analysis was 93.1%, which was very high, and we could proceed to select the desirable genotype. For the GGE biplot analysis, the variability explained by the principal components (PCs) was 100% (PC1: 95.2% and PC2: 4.8%). All genotypes were stable, i.e., they all had a near-stable performance over all environments since they were represented as being nearly parallel on the adaptation map and had a small deviation from the AEA vector on the GGE biplot genotypes view. The desirable genotype over all environments was the G2 (Tanagra) as the most productive on the AMMI1 biplot and the genotype in the concentric area of the ideal genotype on the GGE biplot genotypes view and the which-won-where biplot (Figure 1).

Regarding the thousand-kernel weight, the variability explained by the AMMI analysis was 87.6% and GGE biplot was 99.7% (PC1: 93.7%, PC2: 6%). According to the adaptation map of the AMMI analysis and the GGE biplot of the genotype view, all genotypes appeared to have generally stable performances. The G2 (Tanagra) genotype, which did exceptionally well in the majority of environments, as shown in the which-won-where figure and the AMMI1 and GGE genotypes view biplot, was the most preferred genotype for the thousand-kernel weight. But in the E3 (Trikala) environment, the G1 (Polycarpe) genotype seemed to express a specific adaptability (Supplementary Figure S1e).

For the trait of the number of pods per plant, the variability explained by the AMMI analysis was 67.6% and GGE biplot was 82.1% (PC1: 56.2%, PC2: 25.9%). The genotypes that expressed specific adaptability on this trait were the G2 (Tanagra), followed by the G4 (Ste2), which seemed to be desirable based on the AMMI1 and GGE genotype view for the E1 (Giannitsa), E3 (Trikala), and E4 (Kalambaka) environments. The which-won-where figure shows that the G3 (Ste1) genotype adapted better in the E2 (Florina) environment (Supplementary Figure S2).

For the trait of plant height, the AMMI analysis explained 56.9% of the variability and the GGE biplot explained 98.4% (PC: 94.1% and PC2: 4.3%). All genotypes were shown to be stable across every environment, according to the analysis. This was evident in both the GGE biplot of the genotype view and the AMMI adaption map (Supplementary Figure S3). All genotypes were placed close to the AEA vector and G2 (Tanagra) was placed near the ideal genotype. The G2 (Tanagra) genotype was the most stable and desirable genotype across all environments according to the which-won-where figure (Supplementary Figure S3).

For the trait of earliness, the AMMI analysis explained 72.1% of the variability and the GGE biplot explained 99.7% (PC: 95.2% and PC2: 4.3%). The figures of the adaptation map, AMMI1, and GGE genotype view show that the desirable genotypes were G4 (Ste2) and G2 (Tanagra), which were placed on the right part of AMMI1 biplot and the concentric area of the ideal genotype. The which-won-where plot shows a specific adaptability of the G2 (Tanagra) genotype for the E4 environment, while the G4 (Ste2) genotype was adapted better in the E1 (Giannitsa), E2 (Florina), and E3 (Trikala) environments (Supplementary Figure S4).

**Figure 2:**
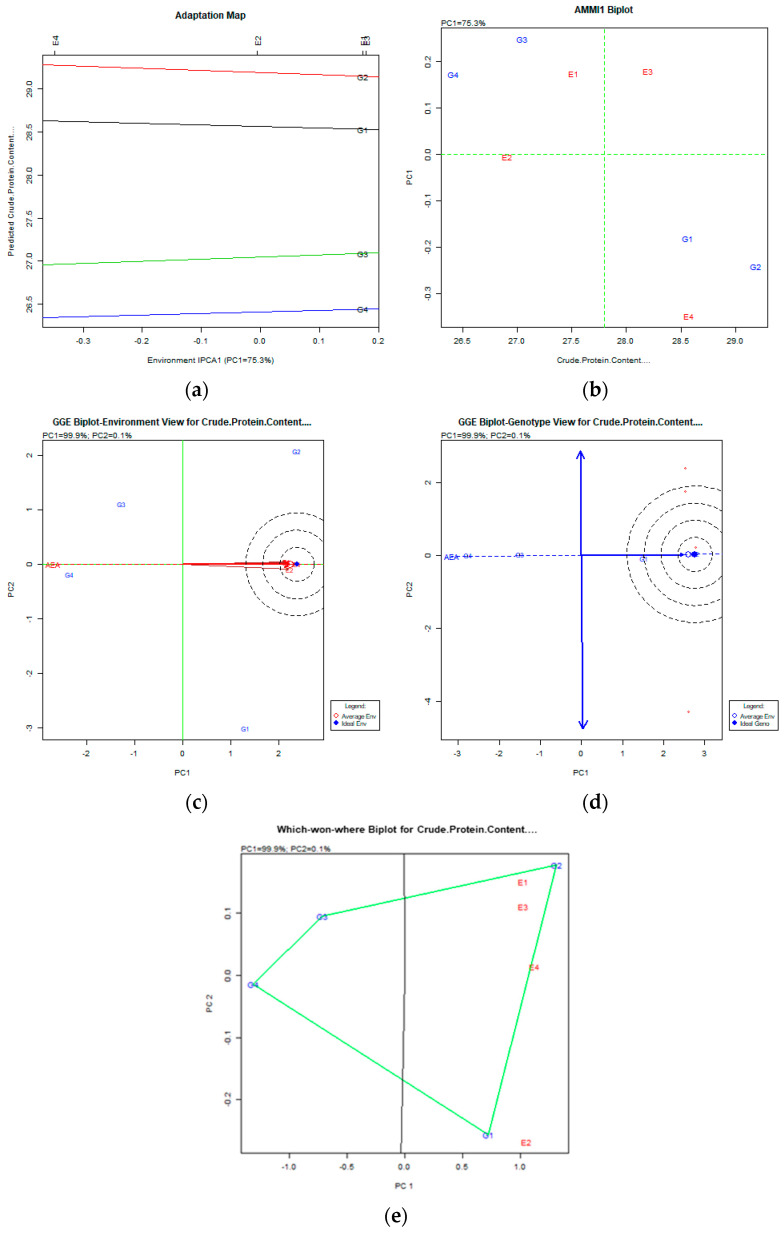
Crude protein content stability analysis based on (**a**) AMMI adaptation map, (**b**) AMMI1 biplot, (**c**) environmental stability GGE biplot, and (**d**) genotypic stability GGE biplot. The genotypes closer to the ideal genotype are more desirable. (**e**) Which-won-where GGE biplot for specific adaptability of genotypes over environments.

For the trait of crude protein, the AMMI analysis explained 75.3% of the variability and the GGE biplot explained 100% (PC: 99.9% and PC2: 0.1%). The AMMI analysis using the adaptation map and AMMI1 biplot, along with the GGE biplot analysis, showed that all genotypes were stable across all environments, whereas the most desirable genotypes were the G2 (Tanagra), along with the G1 (Polycarpe). The which-won-where plot revealed that G1 (Polycarpe) expressed specific adaptability for the E2 (Florina) environment, whereas the G2 (Tanagra) genotype was stable in all other environments (Figure 2).

For the trait of fat content, the AMMI analysis explained 95.0% of the variability and the GGE biplot explained 99.9% (PC: 98.2% and PC2: 1.7%). All genotypes were stable across all environments according to the AMMI analysis using the adaptation map and AMMI1 biplot, along with the GGE biplot analysis, with the desirable genotypes being the G4 (Ste2) and G2 (Tanagra). In the E4 (Kalambaka) environment, the G2 (Tanagra) expressed specific adaptability, while it was dominant in all other environments (Supplementary Figure S5).

For the trait of ash content, the AMMI analysis explained 71.9% of the variability and the GGE biplot explained 98.8% (PC: 94.9% and PC2: 3.9%). The G4 (Ste2) genotype was the desirable genotype according to the AMMI analysis using the adaptation map and AMMI1 biplot combined with the GGE biplot analysis (Supplemental Figure S6). All genotypes were stable across all environments.

The AMMI analysis explained 52.7% of the variability and the GGE biplot explained 89.1% (PC: 71.1% and PC2: 17.2%) for the trait of starch content percentage. The desirable genotypes were G3 (Ste1), followed by G4 (Ste2), based on the AMMI analysis using the adaptation map and AMMI1 biplot, along with the GGE biplot analysis (Supplementary Figure S7).

Regarding the crude fiber content, the AMMI analysis explained 70.2% of the variability and the GGE biplot explained 100% (PC: 99.6% and PC2: 0.4%). The AMMI analysis using the adaptation map and AMMI1 biplot, along with the GGE biplot analysis, showed that all genotypes were stable across all environments and the desirable genotypes were G3 (Ste1), followed by G4 (Ste2). The G4 (Ste2) genotype expressed specific adaptability in the E2 (Florina) environment (Supplementary Figure S8).

The AMMI analysis explained 68.6% of the variability and the GGE biplot explained 86.4% (PC: 50.3% and PC2: 36.1%) for the trait of water content percentage. The AMMI analysis using the adaptation map and AMMI1 biplot, along with the GGE biplot analysis, revealed that the desirable genotype was G4 (Ste2) (Supplementary Figure S9).

## 3. Discussion

In the current study, we employed AMMI plus GGE biplots, ANOVA, stability index computations, seed yield, and chemical composition measurements in faba bean multi-location field experiments to examine the stability and performance of faba bean genotypes (cultivars and selection lines) in various environments and two types of agricultural farming.

According to the ANOVA table, GxE interactions were quite significant in our research, even though earliness and crude protein had a lower significance. In their study of yield-related stability metrics under strong GxE interactions, Tamesgen et al. [13] and Di Paolo et al. [45] discovered a considerable GxE relationship. Additionally, Gurmu et al.’s [46] research on the yield stability of faba beans showed that there were GxE interactions that affected the yield. Mekiso Halengo et al. [20] reported that the influence of the environment on faba bean grain yield was found to be significant when they used the AMMI tool along with Wricke’s stability parameter in order to identify stable and high-yielding genotypes in eight environments.

Detailed data tables showed that earliness, starch content, and plant height all displayed indices over 1000, especially earliness, which reached 4000 in the Trikala area under conventional treatment, and almost reached the same level under low-input treatment, indicating high stability for these traits according to Greveniotis et al. [32,33,35]. Trikala and Kalambaka showed high plant height and seed yield indices, pointing to an environment that is favorable for quantitative traits that ensure yield stability. Ste1 and Tanagra genotypes showed some extreme index values for earliness that were even near or above 5000. For the studied genotypes, earliness demonstrated extreme stability index values in numerous regions. Polycarpe generally showed lower indices because it is an old variety that was developed in different-than-present environmental conditions [14]. The improved and newer cultivar Tanagra displayed unstable behavior and low indices for seed yield in low-input conditions, indicating that breeding improvement was performed under favorable conditions [47], where a high stability index was found for protein content as a result of successful improvement for that trait. Tanagra demonstrated high values for seed yield in Giannitsa and Florina under conventional treatment. This was a result of successful breeding under advantageous conditions as well [47]. The genetic materials Ste1 and Ste2 are very promising, demonstrating high values under low-input conditions.

All qualitative traits showed high heritability values based on the stability index, i.e., over 90 and near 100, except for water content. It is possible that faba beans may incorporate physical adaptability according to climatic conditions. Earliness, although considered a quantitative trait, showed high heritability values and very high stability indices, indicating qualitative behavior [48]. Quantitative traits like seed yield showed relatively low-stability-based heritability values but at a satisfactory level, except for the number of pods per plant, which exhibited low heritability, although this trait showed satisfactory index values. Alan and Geren [49] showed that heritability in faba beans may vary from only 3% for pods per plant, 29% for plant height, 30% for TSW, 47% for seeds per pod, to 77% for seed yield. In faba beans, Toker [11] reported that heritability for plant height was 83%; for the number of pods per plant—43%; for seed yield—62%; and for 100-seed weight, days to flowering, and maturity—over 90%. It was found that the seed weight was the least affected trait across changing environmental conditions, followed by days to flowering and maturity. Kumar et al. [48] reported generally high-to-moderate heritability of quantitative traits and yield components.

Correlations showed that the seed yield was positively correlated with the thousand-seed weight (TSW) and plant height, as well as the crude protein content, but negatively with the rest of the qualitative traits, like starch content. The number of pods per plant seems not to have affected the rest of the qualitative or quantitative traits. It seems that TSW could serve as an indicator of stability in breeding experiments that also reflect a higher and more stable yield. This kind of indirect selection may assist breeders in faster achieving improved genetic materials for the most important characteristics [31,32,33,34]. Alan and Geren [49] reported a positive correlation between the seed yield and seed yield per pod. Ulukan et al. [50] also reported positive correlations between the yield components of faba beans. Most important are the considered correlations of qualitative traits with yield components, where they are useful for indirect selection to accelerate the breeding procedure.

Based on the AMMI and GGE biplot analyses, the most productive and stable genotype across all environments was the G2 (Tanagra) genotype. In our experiments, the seed yield was affected by the different genotypes. Temesgen et al. [13], using the AMMI tool, also managed to define a few genotypes that are adaptable to certain favorable environments. The GGE biplot was the main tool used by Gurmu et al. [46] in order to define the most adaptive cultivars and the most stable environments. It was also used by Haile et al. [41] to assess twelve faba bean genotypes for high mean yield and, subsequently, to identify stable varieties across seven locations. Our data indicated that some genotypes showed specific adaptability in some traits, like the G1 (Polycarpe) genotype in the E3 (Trikala) environment for the thousand–kernel weight, the G3 (Ste1) genotype in the E2 (Florina) environment for the number of pods per plant, the G2 (Tanagra) genotype in the E4 (Kalambaka) environment for earliness, and the G1 (Polycarpe) genotype in the E2 (Florina) environment for the crude protein content. In general, all genotypes expressed stability in all environments. The analysis did not depict very diverse environments. Specific adaptation and stability of performance are useful in areas exhibiting extreme environmental conditions.

Concerning the TSW, since it is highly heritable [11,51], it is considered that adaptation across different environments may result in stable cultivars with satisfactory yields [40], like Tanagra, while Polycarpe showed only specific adaptation. For the number of pods per plant and plant height, the cultivar Tanagra reached the ideal genotype, indicating efficient breeding and good adaptation in most environments. The variation explained was near other researchers’ findings, as reported by Papastylianou et al. [40].

For the trait of earliness, the AMMI analysis explained 72.1% of the variability, while the GGE biplot explained 99.7%. Tanagra was the best genotype in Kalambaka, while the Ste2 genotype was adapted better in Giannitsa, Florina, and E3 Trikala.

For the trait of crude protein, the AMMI analysis explained 75.3% of the variability and the GGE biplot explained 100%. Tanagra and Polycarpe expressed good behavior and adaptation. For the trait of fat content, the AMMI analysis explained 95.0% of the variability and the GGE biplot explained 99.9%. All genotypes were stable across all environments according to the AMMI analysis using the adaptation map and AMMI1 biplot, along with the GGE biplot analysis, where Tanagra again exhibited the best behavior. For the trait of ash content, the AMMI analysis explained 71.9% of the variability and the GGE biplot explained 98.8%. The Ste2 genotype was the desirable genotype according to the AMMI analysis using the adaptation map and AMMI1 biplot combined with the GGE biplot.

Papastylianou et al. [40] reported that GGE biplot analysis for high yield and stability across environments revealed three main genotypic types: genotypes well adapted for biomass or seed yield and genotypes with high adaptation capacity for both traits under Mediterranean conditions. These results indicate that a stability analysis of faba bean genotypes under different environmental conditions is essential to identify adaptable and stable cultivars that are to be cultivated for biomass and seed yield or useful to breeding programs. The GGE biplot better explained our data, and thus, we propose it as a more useful tool to explain the variability and define the ideal genotypes for certain environments.

## 4. Materials and Methods

### 4.1. Establishment of Crops and Experimental Techniques

**Figure 3:**
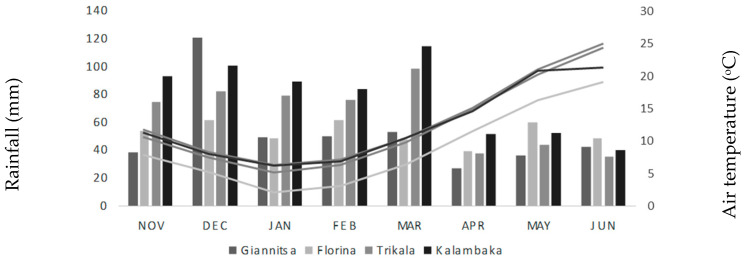
Meteorological data means of mean air temperature (°C) and total monthly rainfall (mm) for the two years of experimentation.

Four separate locations, two in Northern Greece and two in Central Greece, were utilized for the field trials. These locations varied in terms of the soil type, altitude, and environmental variables (Table 7 and Figure 3).

Two commercial cultivars (G1: Polycarpe and G2: Tanagra) and two selection lines (G3: Ste1 and G4: Ste2) from local populations were used as the genetic materials and were cultivated using a specific randomized strip-plot design. Early varieties Polycarpe and Tanagra, which were developed at the Institute of Industrial and Forage Crops (Hellenic Agricultural Organization—Demeter), are resilient to the cold (down to −10 °C), are suitable for autumn sowing, are productive, and adapt to various soils and climates. The genetic materials were selected in order to test the available commercial cultivars, which were developed in our Institute using the selection lines for their stability of yield in varying environments, to compare their characteristics in conventional and low-input conditions, as well as to identify the more stable cultivars across all stability methods.

Within each plot, the four genetic materials were planted at random. Each plot had a total size of 8.75 m^2^, with seven rows that were each five meters in length and separated by 0.25 m. The experiment had four replications and the same design was used in all environments.

The use of traditional and low-input agricultural farming was employed. The standard farming approach treated the plots before sowing, adding 30 and 80 kg ha^−1^ of nitrogen and P_2_O_5_ to the soil, respectively. No fertilizers or other agro-chemicals were utilized in any of the four selected locations throughout the experiment in order to practice low-input farming. Without the use of supplemental nutrients or other agro-chemicals, the fields were previously used to grow bread wheat and legumes in a rotation. Manual weed control was performed in the experimentation area.

### 4.2. Measurements

The following characteristics were measured: seed yield in kilograms per hectare, thousand-seed weight (TSW) in grams, number of pods per plant, plant height in centi-meters, and earliness in days after sowing. Moreover, seed characteristics, such as crude protein (%), crude fat (%), ash (%), total starch (%), crude fiber (%), and moisture (%), were evaluated in the Laboratory of Animal Technology at the University of Thessaly. Samples were ground before the analyses. With regard to the applied methodology, the American Association of Cereal Chemists (AACC) method 44-15.02 [52] for total nitrogen was used, which was then multiplied by a factor of 6.25 to estimate the crude protein content (%). Using the Soxhlet extraction apparatus and petroleum ether extraction, the crude fat (%) was calculated (AACC method 30-25.01) [52]. AACC method 44-15.02 [52], which is an air oven method, was applied to calculate the moisture content (%), while the AACC method 08-01.01 [52], which is a fundamental incineration method, was utilized to calculate the ash content (%) by heating the sample in a furnace to 550 °C until it attained a constant weight, then cooling it in a desiccator and weighing once it reached room temperature. An enzymatic method (AACC method 76-13.01) [52] was used to determine the total amount of starch (%), employing the enzymatic conversion of the α-linked-glucose carbohydrate to glucose and subsequent detection of the released glucose utilizing the Megazyme Amyloglucosidase/alpha-Amylase protocol (Megazyme International Ireland Ltd. Bray, Ireland). The crude fiber (%) was calculated using AACC method 32-10.01 [52], which calls for a sequence of digestions with sulfuric acid and sodium hydroxide, followed by drying, weighing, and igniting of the insoluble residue, and then computation of the crude fiber from the ignition loss.

### 4.3. Data Analysis

Stability estimations were created while incorporating the stability index ${\left(\bar{x}/s\right)}^{2}$, where $\bar{x}$ and *s* stand for the entry mean yield and standard deviation, respectively [31,32,33,34,35,36,37,44,53,54].

ANOVA was used to evaluate significant interactions, the Pearson coefficient to analyze trait correlations, and SPSS version 25 (International Business Machines—IBM Corporation, Chicago, IL, USA) to determine the significance of each statistic at *p* < 0.05, according to Steel et al. [55].

The variance components were calculated using the mean-squared values of the genotypes, genotype × environment, error, and replicates in accordance with McIntosh’s suggestions [56]. This made it possible for us to establish the genetic parameters for the traits under consideration.

According to Johnson et al. [57] and Hanson et al. [58], the heritability in a broad sense (H^2^) was calculated as follows:

$$H_{}^{2}=\frac{\sigma_{g}^{2}}{\sigma_{g}^{2}+\frac{\sigma_{gxe}^{2}{}_{}}{e}+\frac{\sigma_{re}^{2}}{rxe}}$$

According to Singh and Chaudhary [59], the genotypic coefficient of variation (GCV) and the phenotypic coefficient of variation (PCV) were calculated for each evaluated feature:

$$\mathrm{GCV}(\%)=\frac{\sqrt{\sigma_{g}^{2}}}{\bar{x}}\;\times \;100$$

$$\mathrm{PCV}(\%)=\frac{\sqrt{\sigma_{p}^{2}}}{\bar{x}}\;\times \;100$$

where the genotypic variance, phenotypic variance, genotype × environment variance, residual variance (error), and overall mean for every examined attribute are denoted by $\sigma_{g}^{2}$, $\sigma_{p}^{2}$, $\sigma_{gxe}^{2}$, $\sigma_{re}^{2}$, and $\bar{x}$, respectively.

### 4.4. The Multi-Environment Evaluation AMMI Tool

The AMMI analysis is a software tool used for multi-environment analysis in order to depict genotype × environment interaction. The AMMI software creates tables in a two-way GEI. The least squares are estimated and used to produce a two-way ANOVA for an additive model for the main effects and a value to express the residuals interaction [60].

The software utilized was the International Rice Research Institute’s (Laguna, Philippines) PB tools v1.4 free version. This AMMI software tool generates a figure of adaptation map and an AMMI1 biplot with the two axes, where the X-axis represents the factor and the Y-axis represents the PC1 value. When the PC1 value is low, then the distance from the *X*-axis is short, which means that the analyzed factor is stable for all environments.

GGE analysis investigated the genotype main effect (G) combined with the genotype-by-environment interaction (GEI); this made it the main component of variance that was applied in the assessment of genotypes. In mathematics terms, the GGE consisted of the genotype by environment (GxE) data matrix on which the environment means were subtracted. In the two-way data, a GGE biplot depicts the GGE of the genotype-by-environment interaction [28,29].

Using the GGE biplot over environments, the most stable and desirable environment is the one placed near the average and ideal environment; the ideal and desirable genotypes are located in the zone surrounding the average genotype dot and close to the ideal genotype. 

## 5. Conclusions

In a changing environment, the stability of performance has become of greater importance than performance per se. Our innovative approach for estimating stability depended on stability index measurements in faba bean multi-location field experiments by utilizing ANOVA, comparative data, genetic parameter calculations, and AMMI plus GGE biplots tools. This study identified the genotypes that adapted well and uniquely to each environment.

Significant GxE interactions were present for all traits measured. According to the comparative data and ANOVA, Ste1 and Tanagra showed some extreme index values for earliness, followed by Ste2. Earliness showed extreme stability index values in many areas or environments for the genotypes tested, indicating a qualitative trait. For the seed yield, the improved cultivar Tanagra showed unstable behavior and low indices in low-input conditions, indicating that breeding improvement was performed under favorable conditions, but for crude protein content, it showed a high stability index, followed by Ste1. Tanagra showed high values in Giannitsa and Florina under conventional treatment. Ste1 and Ste2 are very promising genetic materials under low-input conditions.

According to the genetic parameters, earliness showed high heritability values and also very high stability indices, again indicating qualitative behavior. Traits like the faba bean seed yield showed relatively low-stability-based heritability values and the number of pods per plant exhibited low heritability, although this trait showed satisfactory index values.

According to the AMMI and GGE biplots, the optimal genotypes to employ and the most favorable environments/types of cultivation were the cultivar Tanagra, followed by the Ste2 genotype.

Correlations showed that the seed yield was positively correlated with the thousand-seed weight (TSW) and plant height, as well as with the crude protein content, but negatively with the rest of the qualitative traits. Thus, indirect selection may assist breeders in more quickly achieving improved genetic materials in the most important characteristics.

As a final conclusion, our combined analysis of stability indices led to the determination of the most stable genotypes and, simultaneously, the most favorable environments. We also propose stability-index-based correlations to serve as a tool for indirect selection that may result in the acceleration of the breeding procedure.

## Figures and Tables

**Table 1:** Mean squares from analysis of variance over environments and cultivation methods for tested traits: seed yield (kg ha^−1^), thousand-seed weight (g), number of pods per plant, plant height (cm), earliness in days after sowing, crude protein content (%) of dry matter, fat content (%) of dry matter, ash content (%) of dry matter, starch content (%) of dry matter, crude fiber content (%) of dry matter, and water content (%).

Source of Variation	Seed Yield (kg ha^−1^)	Thousand-Seed Weight (g)	Number of Pods per Plant	Plant Height (cm)	Earliness in Days after Sowing	Crude Protein Content (%)	Fat Content (%)	Ash Content (%)	Starch Content (%)	Crude Fiber Content (%)	Water Content (%)
	m.s.	m.s.	m.s.	m.s.	m.s.	m.s.	m.s.	m.s.	m.s.	m.s.	m.s.
Environments (E)	281,751.68 **	10,775.216 **	16.828 **	19.745 **	56.750 **	24.388 **	0.195 **	0.543 **	10.457 **	1.574 **	6.683 **
REPS/environments	27,419.51 *	1108.418 *	0.956 *	3.150 ns	0.646 ns	0.101 **	0.001 *	0.001 ns	0.123 ns	0.004 ns	0.015 ns
Varieties (G)	2,248,705.07 **	82,345.910 **	10.324 **	226.543 **	216.978 **	106.553 **	0.249 **	3.150 **	42.459 **	31.691 **	6.691 **
Environments × varieties (G × E)	282,026.37 **	10,649.174 **	7.111 **	15.231 **	1.332 *	0.079 *	0.010 **	0.012 **	1.384 **	0.128 **	1.086 **
Error	16,890.93	694.571	0.341	2.655	0.885	0.050	0.001	0.005	0.098	0.005	0.013

*, **—Statistically significant at *p* < 0.05 and 0.01, respectively; ns—not significant.

**Table 2:** Trait stability index across environments for two farming systems: seed yield (kg ha^−1^), thousand-seed weight (g), number of pods per plant, plant height (cm), earliness in days after sowing, crude protein content (%) of dry matter, fat content (%) of dry matter, ash content (%) of dry matter, starch content (%) of dry matter, crude fiber content (%) of dry matter, and water content (%).

	Environments	Seed Yield (kg ha^−1^)	Thousand-Seed Weight (g)	Number of Pods per Plant	Plant Height (cm)	Earliness in Days after Sowing	Crude Protein Content (%)	Fat Content (%)	Ash Content (%)	Starch Content (%)	Crude Fiber Content (%)	Water Content (%)
Conventional	Giannitsa	56	35	171	691	1861	316	539	242	1880	112	313
	Florina	64	30	193	692	2216	364	524	250	1113	98	277
	Trikala	38	40	141	707	3965	367	558	214	1441	115	289
	Kalambaka	79	61	223	1685	2680	321	143	210	1734	131	290
Low Input	Giannitsa	144	107	154	1591	2116	412	135	258	1883	110	489
	Florina	115	80	394	1047	2658	300	90	280	1142	205	100
	Trikala	113	68	321	2084	3479	424	190	231	1456	119	371
	Kalambaka	332	224	519	1051	2401	355	207	236	1748	109	144
Conventional and Low Input	Giannitsa	80	52	156	902	1874	336	198	206	1653	100	345
	Florina	83	44	256	838	2304	300	142	217	1044	122	148
	Trikala	53	49	186	1070	3060	357	230	191	1313	103	330
	Kalambaka	120	95	312	1299	2127	317	170	185	1544	108	191

**Table 3:** Trait stability index across genotypes for the two farming systems: seed yield (kg ha^−1^), thousand-seed weight (g), number of pods per plant, plant height (cm), earliness in days after sowing, crude protein content (%) of dry matter, fat content (%) of dry matter, ash content (%) of dry matter, starch content (%) of dry matter, crude fiber content (%) of dry matter, and water content (%).

	Genotypes	Seed Yield (kg ha^−1^)	Thousand-Seed Weight (g)	Number of Pods per Plant	Plant Height (cm)	Earliness in Days after Sowing	Crude Protein Content (%)	Fat Content (%)	Ash Content (%)	Starch Content (%)	Crude Fiber Content (%)	Water Content (%)
Conventional	Polycarpe	132	92	175	838	2878	486	144	579	2331	640	445
	Tanagra	183	274	134	2035	4244	543	62	279	2075	953	179
	Ste1	192	88	315	1563	4589	538	106	571	2970	888	247
	Ste2	124	65	155	1555	3409	555	60	529	1945	547	180
Low-input	Polycarpe	120	76	336	1777	3231	663	83	631	2323	285	386
	Tanagra	70	43	208	1652	4367	773	77	323	2106	1004	96
	Ste1	193	138	546	1211	5086	651	93	637	2927	1606	259
	Ste2	171	139	148	1452	4643	547	113	565	1945	2632	150
Conventional & Low-input	Polycarpe	127	84	234	1126	2617	499	105	377	1977	326	420
	Tanagra	61	50	166	1634	3673	540	66	238	1791	436	126
	Ste1	160	86	398	1330	3960	518	100	397	2420	429	244
	Ste2	115	67	143	1378	3374	489	76	384	1701	551	158

**Table 4:** Combined trait stability index across genotypes and environments for the two farming systems: seed yield (kg ha^−1^), thousand-seed weight (g), number of pods per plant, plant height (cm), earliness in days after sowing, crude protein content (%) of dry matter, fat content (%) of dry matter, ash content (%) of dry matter, starch content (%) of dry matter, crude fiber content (%) of dry matter, and water content (%).

	Genotypes	Seed Yield (kg ha^−1^)	Thousand-Seed Weight (g)	Number of Pods per Plant	Plant Height (cm)	Earliness in Days after Sowing	Crude Protein Content (%)	Fat Content (%)	Ash Content (%)	Starch Content (%)	Crude Fiber Content (%)	Water Content (%)
Giannitsa												
Conventional	Polycarpe	401	355	107	5374	2269	686	966	1062	3719	416	409
	Tanagra	739	689	958	8975	2475	489	1497	946	3577	841	958
	Ste1	213	160	1415	753	4355	619	2280	960	3336	1473	457
	Ste2	98	49	913	881	3400	745	1017	1172	4102	451	716
Low input	Polycarpe	177	97	428	2733	2201	911	831	821	3604	7619	21,635
	Tanagra	829	1513	108	6211	3049	1482	710	1060	3481	10,536	2605
	Ste1	602	325	505	12,987	4387	1012	1937	1059	3252	5166	1368
	Ste2	122	100	450	9131	3861	826	818	1167	3945	5441	210
Conventional and low input	Polycarpe	260	163	163	2299	2110	758	681	470	2953	390	827
	Tanagra	702	608	198	4493	2649	646	382	521	2820	387	768
	Ste1	123	72	670	1525	3977	672	1957	540	2709	675	625
	Ste2	87	53	127	440	3496	666	302	589	3155	548	293
Florina												
Conventional	Polycarpe	242	136	536	1048	2670	767	1929	800	3969	9552	2064
	Tanagra	1044	519	242	1536	2788	996	956	645	2843	993	1325
	Ste1	213	87	638	11,084	4308	731	2570	874	3527	1496	165
	Ste2	387	285	89	3143	4967	534	767	999	3330	1467	23,467
Low input	Polycarpe	43	28	589	1283	4207	665	628	952	3839	195	233
	Tanagra	426	301	1260	1605	3262	901	90	709	2962	9873	72
	Ste1	208	340	593	2423	4735	820	282	935	3429	1247	250
	Ste2	157	203	677	2901	7004	504	77	1172	3185	22,974	85
Conventional and low input	Polycarpe	78	50	594	801	3145	548	989	474	3061	352	445
	Tanagra	129	71	423	1611	3027	698	143	434	2379	615	146
	Ste1	138	79	617	4077	4166	671	524	500	2839	330	204
	Ste2	160	111	118	2377	5281	501	116	601	2666	684	161
Trikala												
Conventional	Polycarpe	44	32	215	3000	5204	590	1154	821	3217	984	629
	Tanagra	102	580	88	2757	7005	957	876	654	3192	1616	2268
	Ste1	375	120	809	3615	12,111	648	561	944	4346	946	856
	Ste2	164	111	95	4395	6922	812	1030	825	3884	1924	1913
Low input	Polycarpe	515	334	1509	11,406	3135	1142	304	949	3136	11,010	698
	Tanagra	372	168	487	856	7330	1472	920	819	3058	12,544	752
	Ste1	90	72	1334	7241	7200	959	1440	1057	4144	24,018	1026
	Ste2	776	552	1821	11,629	7744	1002	645	769	3755	50,874	794
Conventional and low input	Polycarpe	87	62	314	4181	3224	656	371	488	2607	434	650
	Tanagra	24	26	129	1060	5314	870	304	474	2568	695	1159
	Ste1	155	96	813	2073	6270	671	714	562	3307	494	961
	Ste2	113	69	142	6786	4997	737	250	507	3056	752	922
Kalambaka												
Conventional	Polycarpe	299	193	324	1526	6917	509	410	839	3234	728	1541
	Tanagra	131	188	254	2063	22,829	835	598	818	3930	1010	336
	Ste1	268	159	205	2624	15,935	784	258	1039	4085	1281	21,494
	Ste2	142	74	644	12,381	9478	817	813	1156	3957	2929	39,380
Low input	Polycarpe	172	113	311	1361	5437	771	290	914	3154	6250	244
	Tanagra	408	374	1838	4353	4815	979	383	906	3782	401	260
	Ste1	502	284	849	416	8523	825	541	1127	3960	12,136	376
	Ste2	439	323	1066	5802	6930	837	423	1343	3904	19,886	227
Conventional and low input	Polycarpe	208	142	332	1501	3964	585	280	449	2630	385	316
	Tanagra	83	109	477	2810	5022	773	457	448	3022	340	258
	Ste1	319	155	227	768	5875	707	364	540	3184	563	564
	Ste2	230	123	846	6631	5146	713	556	611	3063	2330	357

**Table 5:** Estimations of genetic parameters for tested traits: seed yield (kg ha^−1^), thousand-seed weight (g), number of pods per plant, plant height (cm), earliness in days after sowing, crude protein content (%) of dry matter, fat content (%) of dry matter, ash content (%) of dry matter, starch content (%) of dry matter, crude fiber content (%) of dry matter, and water content (%).

Traits	Min	Max	Mean	sd	$\sigma_{g}^{2}$	$\sigma_{p}^{2}$	GCV (%)	PCV (%)	H^2^ (%)
Seed yield (kg ha^−1^)	2177.00	3908.00	2828.16	327.87	30,729.356	35,136.018	6.198	6.628	87.5
Thousand-seed weight (g)	314.00	601.00	457.85	63.90	1120.2615	1286.6548	7.310	7.834	87.1
Number of pods per plant	19.00	26.90	23.04	1.65	0.0502	0.1613	0.973	1.743	31.1
Plant height (cm)	82.70	97.10	89.95	2.93	3.3018	3.5397	2.020	2.092	93.3
Earliness in days after sowing	112.10	125.30	118.91	2.60	3.3695	3.3903	1.544	1.549	99.4
Crude protein content (%)	23.93	31.64	27.80	1.66	1.6637	1.6649	4.639	4.641	99.9
Fat content (%)	0.90	1.43	1.08	0.13	0.0037	0.0039	5.681	5.799	96.0
Ash content (%)	2.96	4.31	3.54	0.27	0.0490	0.0492	6.247	6.259	99.6
Starch content (%)	39.36	45.76	42.93	1.20	0.6412	0.6634	1.866	1.897	96.7
Crude fiber content (%)	5.80	8.73	7.17	0.70	0.4932	0.4952	9.797	9.817	99.6
Water content (%)	9.03	13.19	10.73	0.82	0.0876	0.1045	2.759	3.015	83.8

sd—standard deviation, $\sigma_{g}^{2}$—genotypic variance, $\sigma_{p}^{2}$—phenotypic variance, GCV—genotypic coefficient of variation, PCV—phenotypic coefficient of variation, and H^2^—broad sense heritability (%).

**Table 6:** Correlations between all the traits measured: seed yield (kg ha^−1^), thousand-seed weight (g), number of pods per plant, plant height (cm), earliness in days after sowing, crude protein content (%) of dry matter, fat content (%) of dry matter, ash content (%) of dry matter, starch content (%) of dry matter, crude fiber content (%) of dry matter, and water content (%).

	Seed Yield (kg ha^−1^)	Thousand-Seed Weight (g)	Number of Pods per Plant	Plant Height (cm)	Earliness in Days after Sowing	Crude Protein Content (%)	Fat Content (%)	Ash Content (%)	Starch Content (%)	Crude Fiber Content (%)
Thousand-seed weight (g)	0.938 **									
Number of pods per plant	0.020	0.059								
Plant height (cm)	0.359 **	0.331 **	−0.075							
Earliness in days after sowing	0.022	−0.005	−0.072	−0.036						
Crude protein content (%)	0.291 **	0.258 **	−0.099	0.261 **	0.178 **					
Fat content (%)	0.029	0.020	0.132 *	0.090	0.198 **	0.233 **				
Ash content (%)	−0.254 **	−0.283 **	−0.040	−0.264 **	0.633 **	−0.155 *	0.033			
Starch content (%)	−0.319 **	−0.293 **	0.201 **	−0.375 **	−0.107	−0.143 *	0.073	0.255 **		
Crude fiber content (%)	−0.345 **	−0.415 **	−0.030	−0.358 **	0.153 *	−0.534 **	−0.109	0.498 **	0.150 *	
Water content (%)	0.063	0.116	−0.013	0.123 ^*^	0.111	−0.340^**^	−0.224 **	−0.081	−0.629 **	−0.044

* Correlation was significant at the 0.05 level (2-tailed), ** correlation was significant at the 0.01 level (2-tailed).

**Table 7:** Coordinates, elevation, soil type, and cultivation dates for the experimental sites.

Environments	Elevation (m)	Longitude	Latitude	Soil Texture	Planting Date	Harvesting Date
E1: Giannitsa	10	22°39′ E	40°77′ N	Clay	Middle November 2008 and middle November 2009	Late June 2009 and late June 2010
E2: Florina	705	21°22′ E	40°46′ N	Sandy loam	Middle November 2008 and middle November 2009	Late June 2009 and late June 2010
E3: Trikala	120	21°64′ E	39°55′ N	Sandy clay loam	Middle November 2008 and middle November 2009	Late June 2009 and late June 2010
E4: Kalambaka	190	21°65′ E	39°64′ N	Silty clay	Middle November 2008 and middle November 2009	Late June 2009 and late June 2010

## Data Availability

The datasets utilized in this study’s analysis are available upon reasonable request.

## Supplementary Materials

The following supporting information can be downloaded from https://www.mdpi.com/article/10.3390/plants12213769/s1. Supplementary Figure S1: Thousand-seed weight (g) stability analysis based on (a) AMMI adaptation map, (b) AMMI1 biplot, (c) environmental stability GGE biplot, and (d) genotypic stability GGE biplot. The genotypes closer to the ideal genotype are the most desirable. (e) Which-won-where GGE biplot for specific adaptability of genotypes over environments. Supplementary Figure S2: Number of pods per plant stability analysis based on (a) AMMI adaptation map, (b) AMMI1 biplot, (c) environmental stability GGE biplot, and (d) genotypic stability GGE biplot. The genotypes closer to the ideal genotype are the most desirable. (e) Which-won-where GGE biplot for specific adaptability of genotypes over environments. Supplementary Figure S3: Plant height (cm) stability analysis based on (a) AMMI adaptation map, (b) AMMI1 biplot, (c) environmental stability GGE biplot, and (d) genotypic stability GGE biplot. The genotypes closer to the ideal genotype are the most desirable. (e) Which-won-where GGE biplot for specific adaptability of genotypes over environments. Supplementary Figure S4: Earliness in days after sowing stability analysis based on (a) AMMI adaptation map, (b) AMMI1 biplot, (c) environmental stability GGE biplot, and (d) genotypic stability GGE biplot. The genotypes closer to the ideal genotype are the most desirable. (e) Which-won-where GGE biplot for specific adaptability of genotypes over environments. Supplementary Figure S5: Fat content (%) stability analysis based on (a) AMMI adaptation map, (b) AMMI1 biplot, (c) environmental stability GGE biplot, and (d) genotypic stability GGE biplot. The genotypes closer to the ideal genotype are the most desirable. (e) Which-won-where GGE biplot for specific adaptability of genotypes over environments. Supplementary Figure S6: Ash content (%) stability analysis based on (a) AMMI adaptation map, (b) AMMI1 biplot, (c) environmental stability GGE biplot, and (d) genotypic stability GGE biplot. The genotypes closer to the ideal genotype are the most desirable. (e) Which-won-where GGE biplot for specific adaptability of genotypes over environments. Supplementary Figure S7: Starch content (%) stability analysis based on (a) AMMI adaptation map, (b) AMMI1 biplot, (c) environmental stability GGE biplot, and (d) genotypic stability GGE biplot. The genotypes closer to the ideal genotype are the most desirable. (e) Which-won-where GGE biplot for specific adaptability of genotypes over environments. Supplementary Figure S8: Crude fiber content (%) stability analysis based on (a) AMMI adaptation map, (b) AM-MI1 biplot, (c) environmental stability GGE biplot, and (d) genotypic stability GGE biplot. The genotypes closer to the ideal genotype are the most desirable. (e) Which-won-where GGE biplot for specific adaptability of genotypes over environments. Supplementary Figure S9: Water content (%) stability analysis based on (a) AMMI adaptation map, (b) AMMI1 biplot, (c) environmental stability GGE biplot, and (d) genotypic stability GGE biplot. The genotypes closer to the ideal genotype are the most desirable. (e) Which-won-where GGE biplot for specific adaptability of genotypes over environments.
