# Supplementary material for: Genotype-by-Environment Interaction Analysis for Quantity and Quality Traits in Faba Beans Using AMMI, GGE Models, and Stability Indices
Source: Plants (Basel). 2023 Nov 4;12(21):3769. doi: 10.3390/plants12213769 (PMC10648669; doi:10.3390/plants12213769)
Supplement: Supplementary file 1 [file plants-12-03769-s001.zip › plants-2657553-supplementary.pdf]

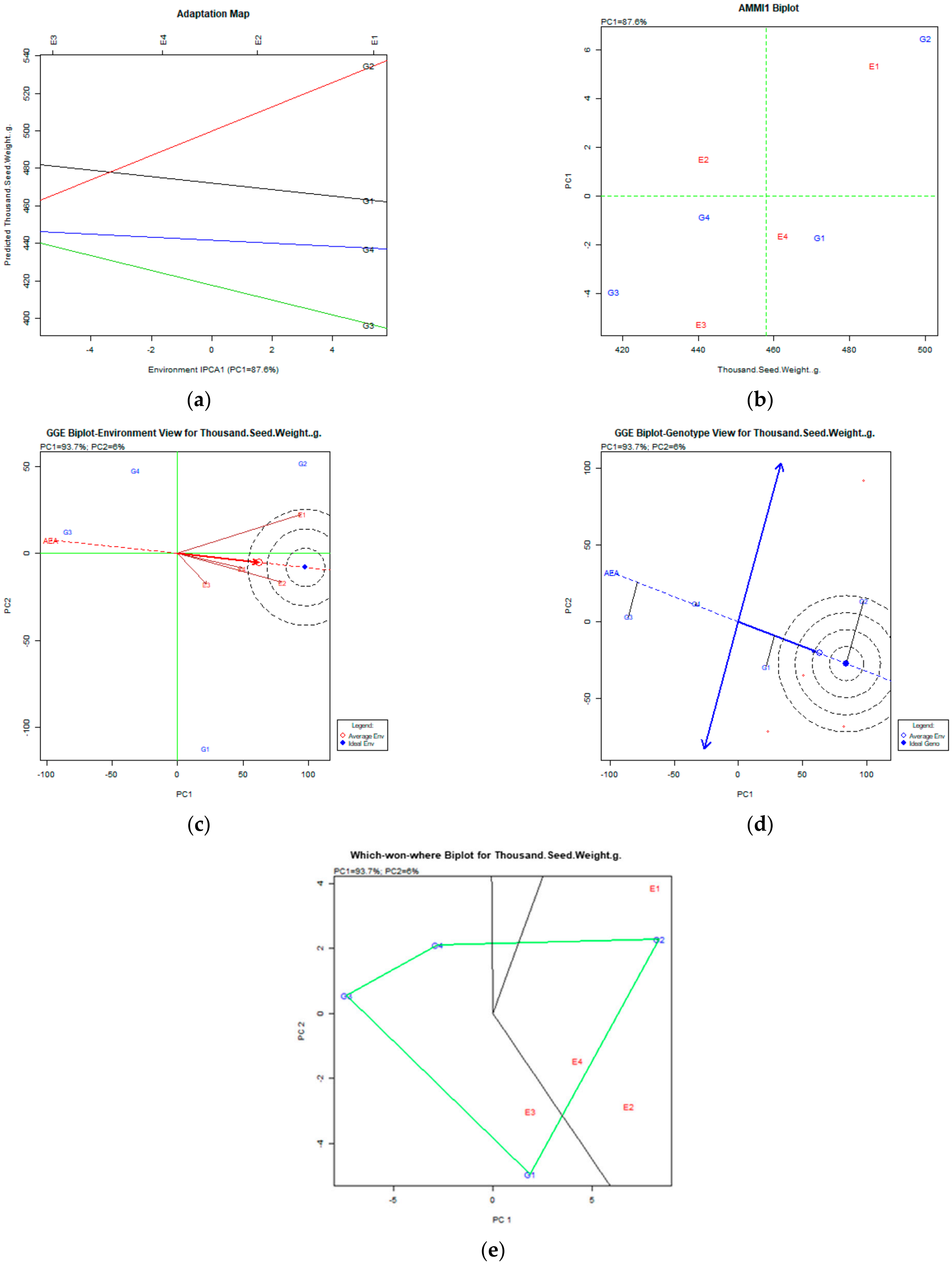

Supplementary Figure S1. Thousand seed weight (g) stability analysis based on (a) AMMI adaptation map; (b) AMMI1 biplot; (c) Environmental stability GGE biplot; (d) Genotypic stability GGE biplot. The genotypes closer to the ideal genotype are the desirable. (e) Which-won-where GGE biplot for specific adaptability of genotypes over environments.

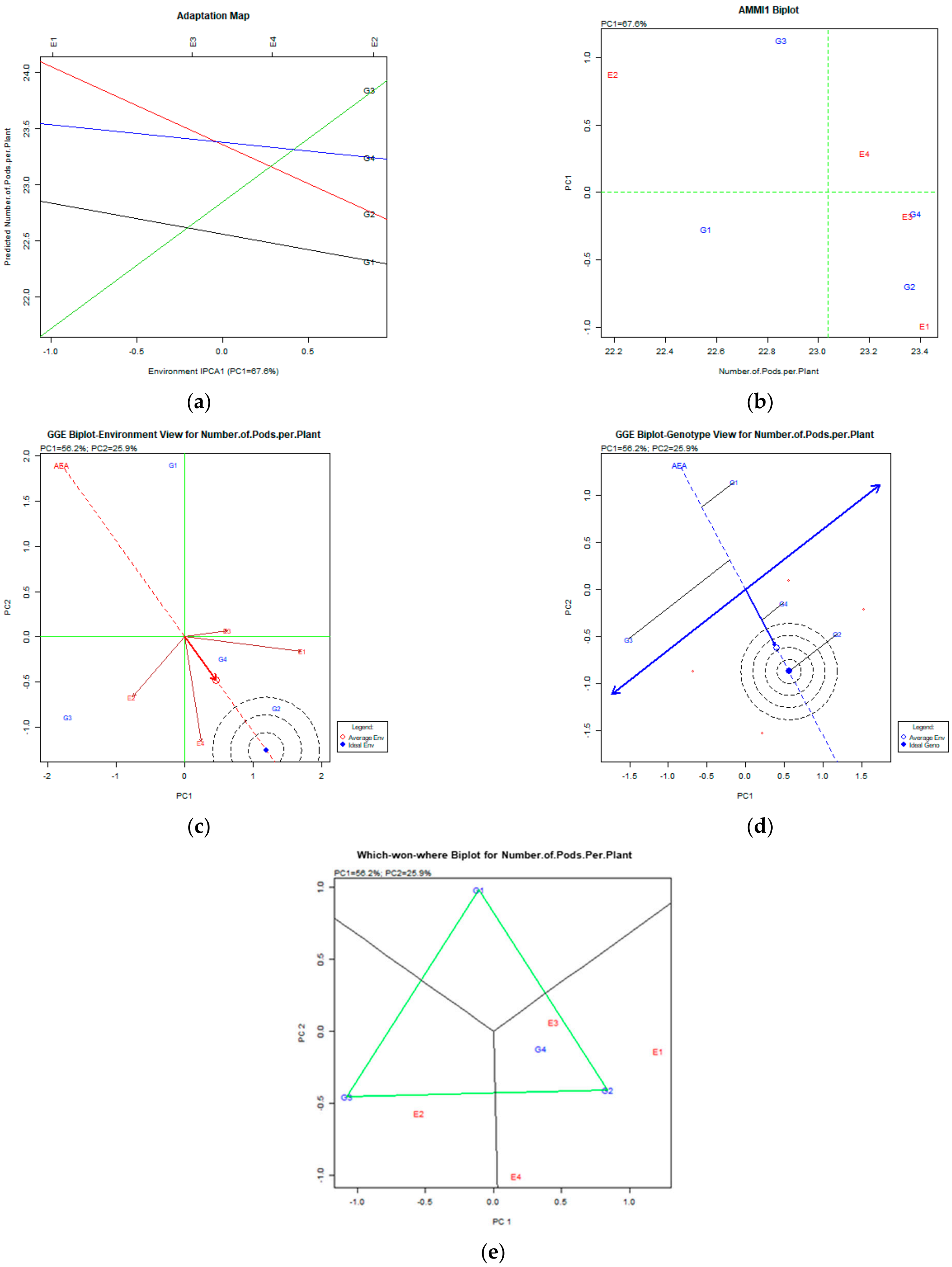

Supplementary Figure S2. Number of pods per plant stability analysis based on (a) AMMI adaptation map; (b) AMMI1 biplot; (c) Environmental stability GGE biplot; (d) Genotypic stability GGE biplot. The genotypes closer to the ideal genotype are the desirable. (e) Which-won-where GGE biplot for specific adaptability of genotypes over environments.

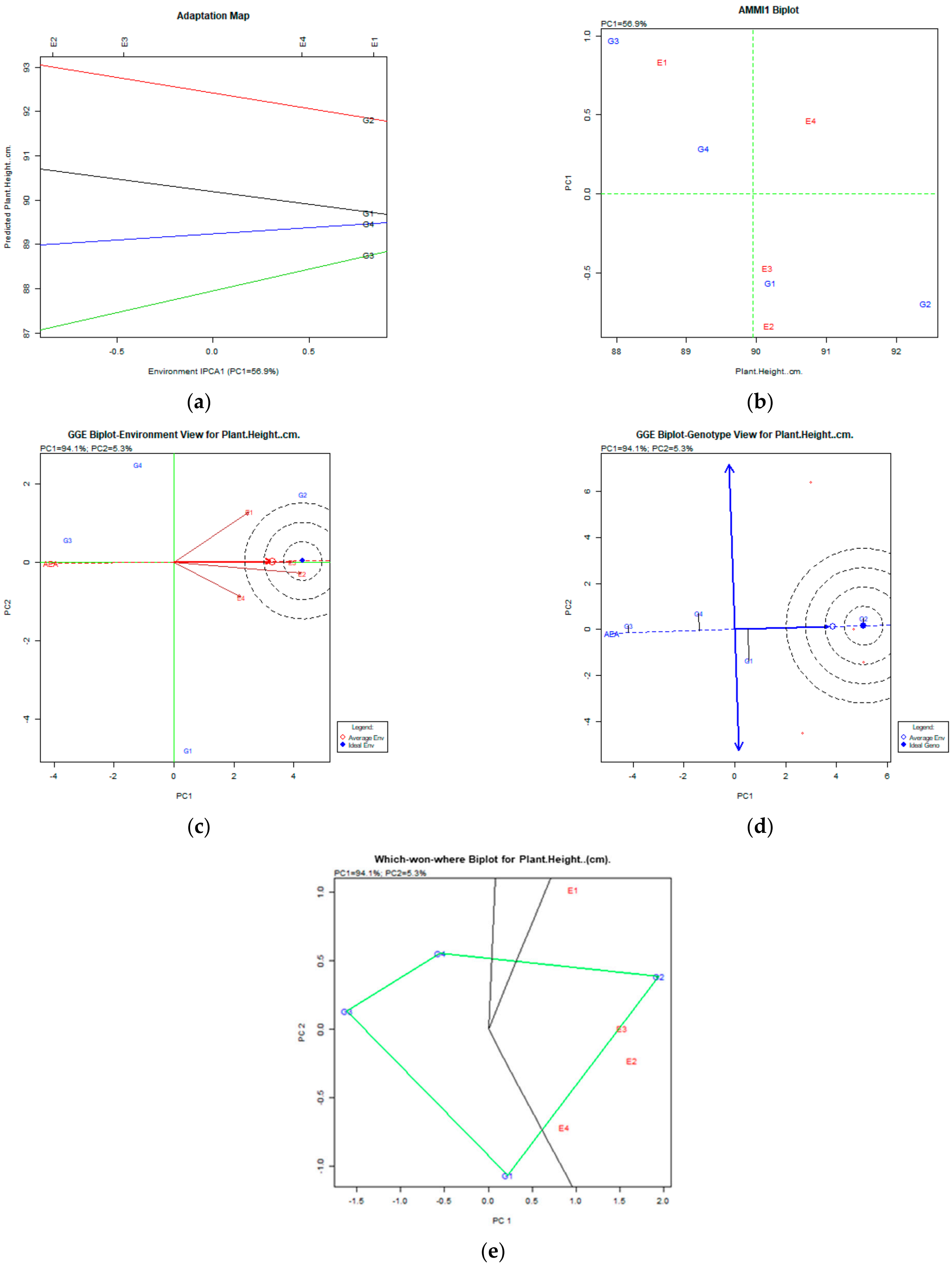

Supplementary Figure S3. Plant height (cm) stability analysis, based on (a) AMMI adaptation map; (b) AMMI1 biplot; (c) Environmental stability GGE biplot; (d) Genotypic stability GGE biplot. The genotypes closer to the ideal genotype are the desirable. (e) Which-won-where GGE biplot for specific adaptability of genotypes over environments.

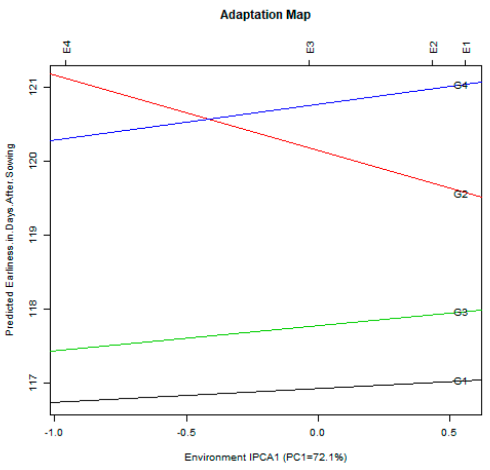

(**a**)

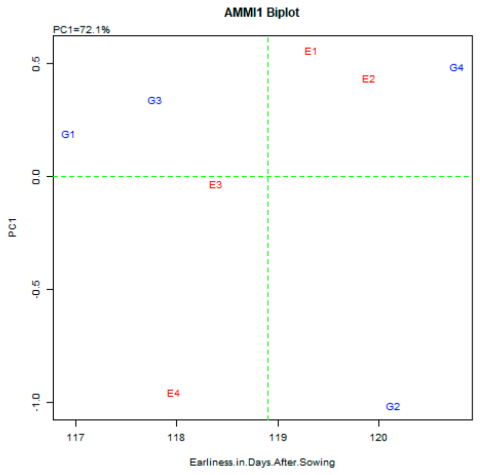

(**b**)

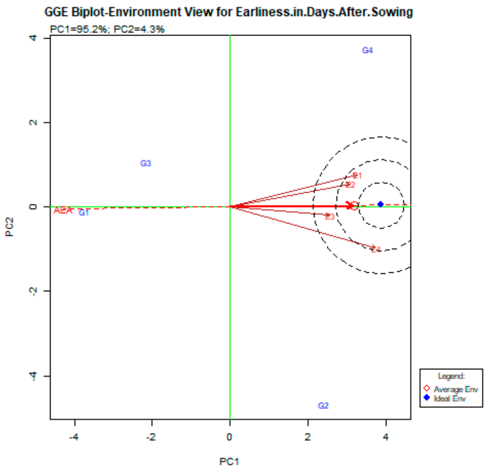

(**c**)

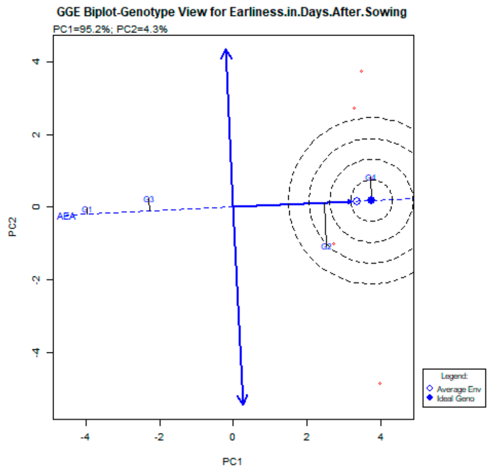

(**d**)

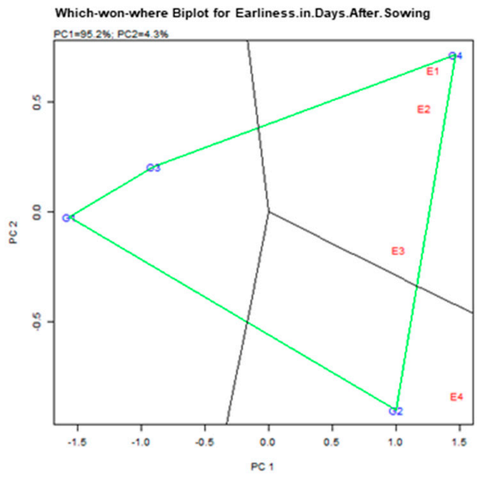

(**e**)

Supplementary Figure S4. Earliness in days after sowing stability analysis based on (a) AMMI adaptation map; (b) AMMI1 biplot; (c) Environ-mental stability GGE biplot; (d) Genotypic stability GGE biplot. The genotypes closer to the ideal genotype are the desirable. (e) Which-won-where GGE biplot for specific adaptability of genotypes over environ-ments.

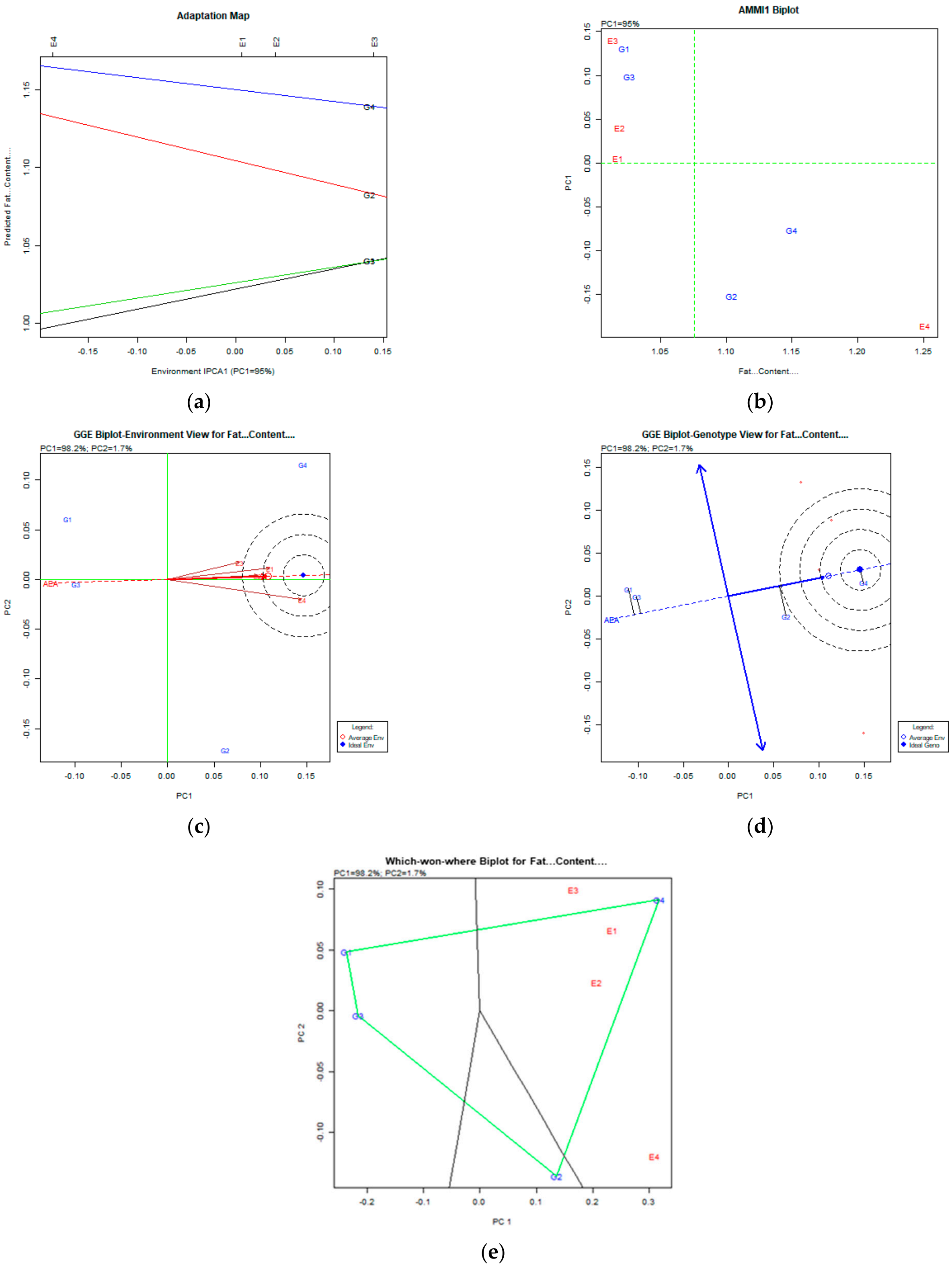

Supplementary Figure S5. Fat content (%) stability analysis, based on (a) AMMI adaptation map; (b) AMMI1 biplot; (c) Environmental stability GGE biplot; (d) Genotypic stability GGE biplot. The genotypes closer to the ideal genotype are the desirable. (e) Which-won-where GGE biplot for specific adaptability of genotypes over environments.

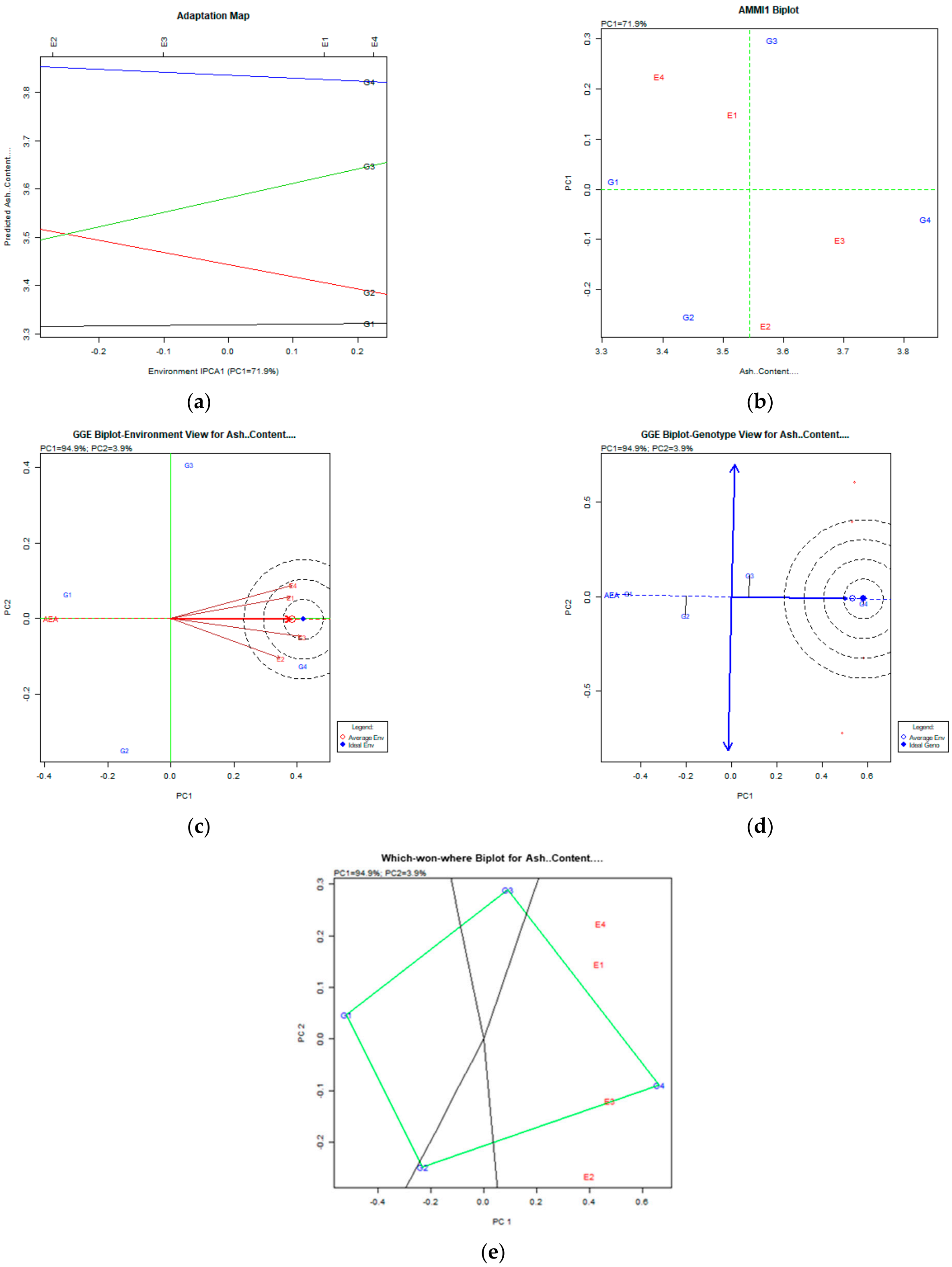

Supplementary Figure S6. Ash content (%) stability analysis based on (a) AMMI adaptation map; (b) AMMI1 biplot; (c) Environmental stability GGE biplot; (d) Genotypic stability GGE biplot. The genotypes closer to the ideal genotype are the desirable. (e) Which-won-where GGE biplot for specific adaptability of genotypes over environments.

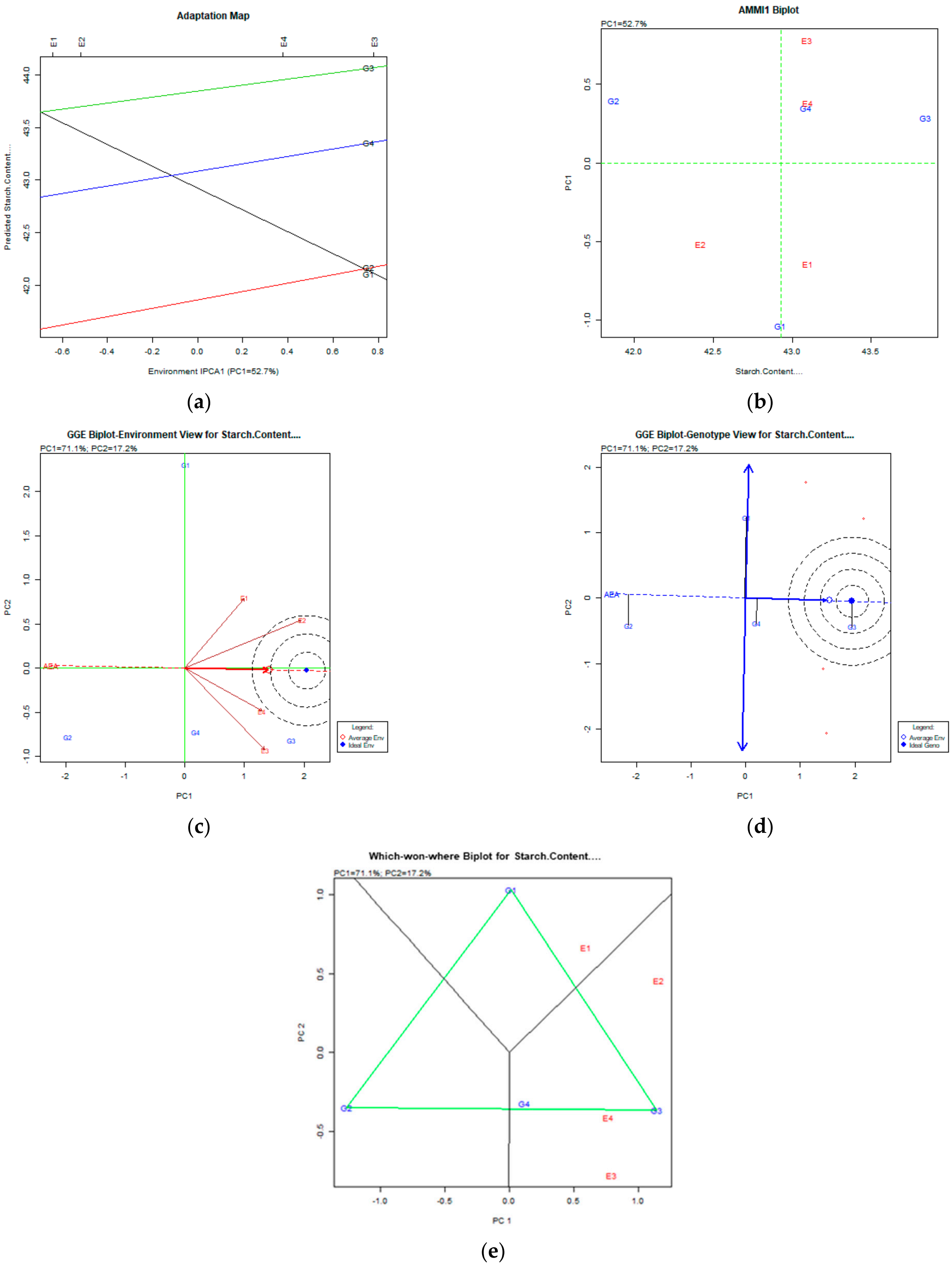

Supplementary Figure S7. Starch content (%) stability analysis based on (a) AMMI adaptation map; (b) AMMI1 biplot; (c) Environmental stability GGE biplot; (d) Genotypic stability GGE biplot. The genotypes closer to the ideal genotype are the desirable. (e) Which-won-where GGE biplot for specific adaptability of genotypes over environments.

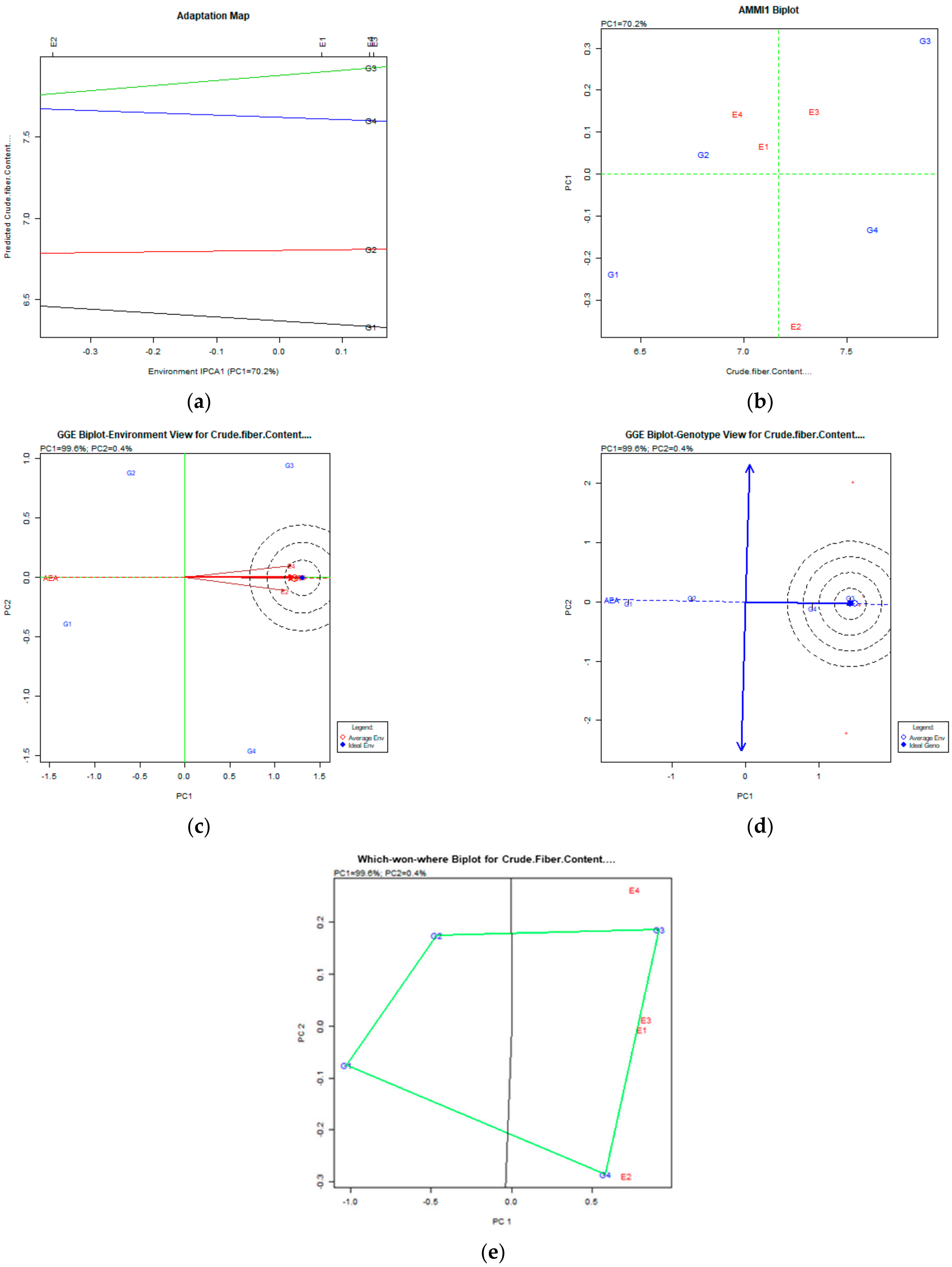

Figure Supplementary S8. Crude fiber content (%) stability analysis based on (a) AMMI adaptation map; (b) AMMI1 biplot; (c) Environmental stability GGE biplot; (d) Genotypic stability GGE biplot. The genotypes closer to the ideal genotype are the desirable. (e) Which-won-where GGE biplot for specific adaptability of genotypes over environments.

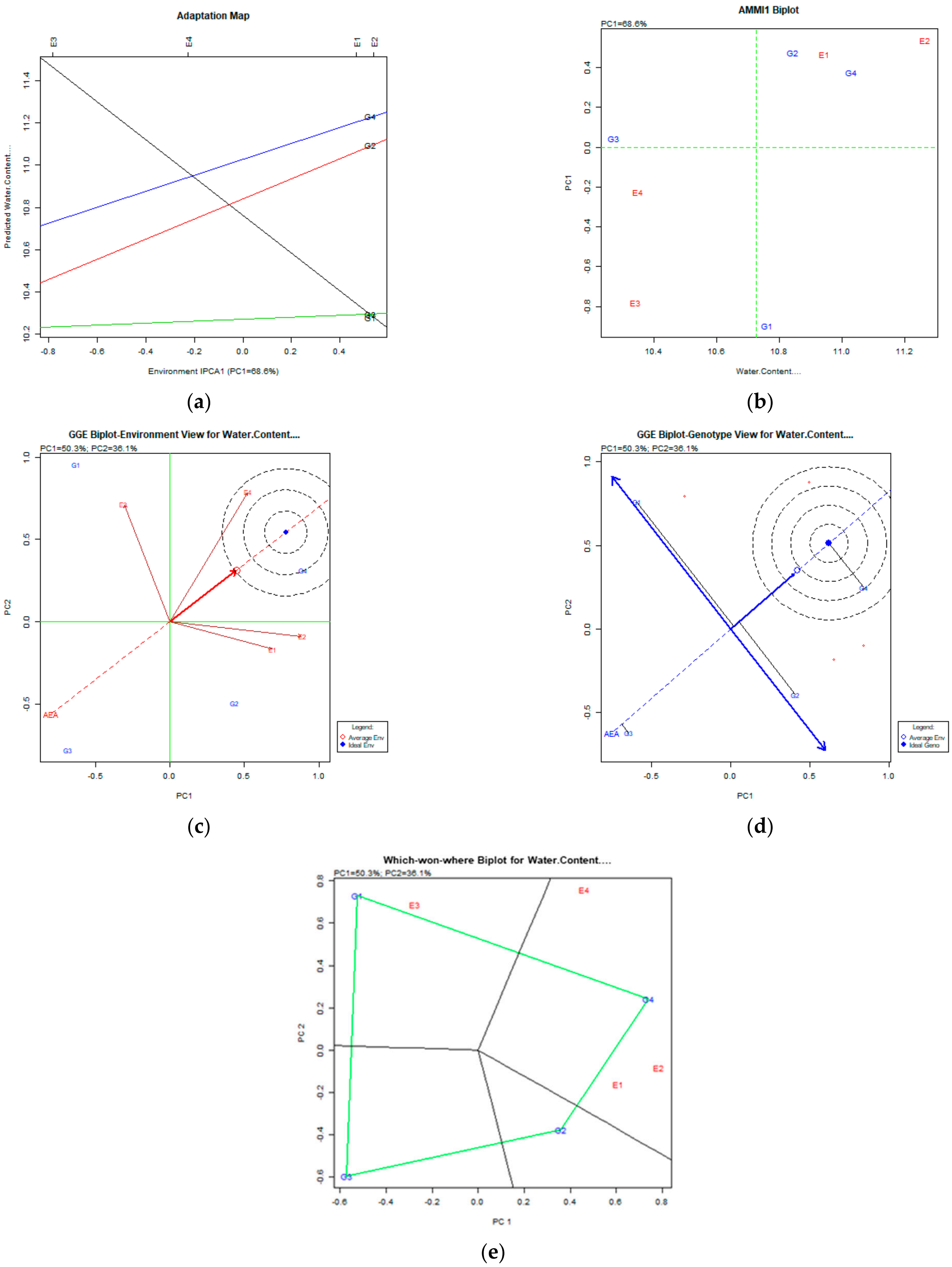

Supplementary Figure S9. Water content (%) stability analysis based on (a) AMMI adaptation map; (b) AMMI1 biplot; (c) Environmental stability GGE biplot; (d) Genotypic stability GGE biplot. The genotypes closer to the ideal genotype are the desirable. (e) Which-won-where GGE biplot for specific adaptability of genotypes over environments.
